# Kinetically Controlled Direct In Situ Photolithography of Perovskite Color‐Conversion Layers

**DOI:** 10.1002/smll.74360

**Published:** 2026-07-03

**Authors:** Mingyu Hwang, Jaedong Jang, Jun‐Seo Lee, Seongkyu Maeng, Sun Jae Park, Seungwoo Lee, Jae Jun Lee, Jong Hui Choi, Jeung Ku Kang, Hyunsu Cho, Himchan Cho

**Affiliations:** ^1^ Graduate School of Semiconductor Technology School of Electrical Engineering Korea Advanced Institute of Science and Technology (KAIST) Yuseong‐gu Daejeon Republic of Korea; ^2^ Department of Materials Science and Engineering Korea Advanced Institute of Science and Technology (KAIST) Yuseong‐gu Daejeon Republic of Korea; ^3^ School of Materials Science and Engineering Yeungnam University Gyeongsan Republic of Korea; ^4^ Reality Display Research Section Electronics and Telecommunications Research Institute (ETRI) Daejeon Republic of Korea

**Keywords:** color‐conversion layer, crosslinking kinetic, direct in situ photolithography, high‐resolution patterning, metal halide perovskites

## Abstract

Metal halide perovskites (MHPs) are promising materials for color‐conversion layers (CCLs) owing to their high absorption coefficients and narrow emission bandwidths. However, high‐resolution patterning of micrometer‐thick MHP CCLs remains challenging due to their susceptibility to optical and chemical damage during conventional patterning processes. Direct in situ photolithography has emerged as a potential solution by enabling high‐thickness pattern formation without directly exposing MHP emitters to harsh conditions, yet its achievable spatial resolution has remained limited. Here, we report a kinetically controlled direct in situ photolithography (KC‐ISP) approach for high‐resolution MHP CCLs. By controlling thiol–ene crosslinking kinetics and incorporating a precursor‐redistributing spin‐washing step, KC‐ISP enables high‐resolution patterning with feature sizes down to ∼3 µm while providing facile color tunability through controlled precursor composition. In addition, in situ crystallized MHPs exhibit a pronounced increase in photoluminescence quantum yield upon air exposure, attributed to phase redistribution and surface defect passivation under ambient conditions. As a result, our MHP CCLs with ∼4 µm thickness achieve blue uptake values of ∼80% and ∼98% for green and red emitters, respectively, with corresponding color‐conversion efficiencies of ∼45% and ∼30%, demonstrating the potential of KC‐ISP for high‐resolution micro‐display applications.

## Graphical abstract

Kinetically controlled direct in situ photolithography (KC‐ISP) enables high‐resolution patterning of micrometer‐thick perovskite color‐conversion layers. By regulating thiol–ene reaction kinetics and redistributing precursors within the polymer matrix, lateral curing is suppressed and spatially selective crystallization is achieved. The resulting films exhibit ∼3 µm feature sizes, efficient color tunability, and high color‐conversion efficiency at only ∼4 µm thickness.

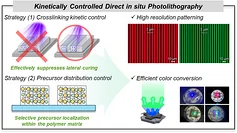

## 1 Introduction

Metal halide perovskites (MHPs) have attracted considerable attention as next‐generation light‐emitting materials owing to their high photoluminescence (PL) quantum yield (PLQY), wide bandgap tunability enabled by compositional engineering, and narrow emission spectra [1, 2, 3, 4]. In particular, MHPs typically exhibit narrow emission linewidths with full width at half maximum (FWHM) down to 25 nm, which enables high color purity compared to InP‐based core/shell quantum dots (QDs) [5, 6]. These optical characteristics have motivated extensive research interest in MHPs as light‐emitting materials for next‐generation display applications [7, 8].

In addition to their favorable emissive properties, MHPs also exhibit intrinsically high absorption coefficients of 10^4^–10^5^ cm^−1^ in the blue spectral range (∼450 nm), enabling efficient harvesting of incident excitation light [9, 10]. These unique optical characteristics make MHPs well suited for color‐conversion layers (CCLs), where efficient absorption of excitation light and its conversion into narrowband emission are required to suppress residual blue‐light leakage and maintain high color purity [9, 10, 11, 12].

Conventional II–VI (e.g., Cd‐based) and III–V (e.g., InP‐based) QDs, however, typically exhibit relatively lower absorption coefficients, which often leads to CCL thicknesses of 7‐10 µm to sufficiently suppress blue‐light leakage [8, 13]. Such thick layers impose substantial challenges for fine patterning below 5 µm, thereby limiting their applicability in high‐resolution micro‐display technologies. By contrast, the intrinsically high absorption coefficients of MHPs enable efficient color conversion within substantially thinner layers, alleviating the thickness–resolution trade‐off encountered in QD‐based systems [14, 15]. Consequently, MHP emitters represent a promising material platform for realizing efficient and high‐resolution color‐conversion layers in next‐generation micro‐displays.

In CCL‐based micro‐displays, precise pixel‐level patterning is required to define individual subpixels and suppress optical crosstalk. While MHPs exhibit promising optical properties, they possess a predominantly ionic lattice characterized by relatively low formation energies for halide vacancies and weak ionic bonding interactions. These intrinsic structural features render MHPs susceptible to defect formation and ion migration under external stimuli [16, 17]. During conventional photolithography processes, exposure to ultraviolet (UV) irradiation, polar solvents, and thermal treatment can readily induce halide migration, vacancy generation, and phase instability. As a result, achieving high‐resolution patterning while preserving the intrinsic optical properties of MHPs remains a central challenge for their integration into micro‐display technologies [18, 19].

To address this challenge, a variety of patterning strategies have been explored, such as transfer printing [20, 21], inkjet printing [22, 23], and direct optical lithography [24, 25, 26, 27, 28, 29]. Among these approaches, direct in situ photolithography has emerged as a promising route for CCLs, as it allows micrometer‐thick patterning while preserving the optical properties of MHPs by avoiding direct exposure to harsh lithographic conditions [30, 31, 32, 33]. In this approach, the photocatalytic activity of PbBr_4_^2−^ complexes facilitates the thiol–ene crosslinking reaction, enabling reduced exposure dose. Despite this progress, the attainable spatial resolution has remained limited to feature sizes on the order of ∼20 µm. This limitation suggests that, beyond photocatalysis, additional chemical parameters governing network formation and pattern evolution play a critical role in defining lithographic fidelity. In particular, the influence of alkene moiety chemistry on thiol–ene crosslinking kinetics, and its subsequent impact on exposure dose, pattern morphology, and resolution has not been systematically investigated.

In this study, we report a kinetically controlled direct in situ photolithography (KC‐ISP) strategy to overcome the resolution limitation of MHP CCLs. We identify thiol–ene reaction kinetics as a decisive chemical factor governing the resolution and fidelity of patterns, and demonstrate that exploiting this kinetic control enables high‐resolution MHP CCLs with high color‐conversion efficiency (CCE). Beyond kinetic control of polymer network formation, the KC‐ISP process further incorporates a precursor‐redistributing spin‐washing step that reconfigures the spatial distribution of MHP precursors within the polymer matrix, thereby improving pattern resolution while providing emission color tunability through controlled variation of the precursor composition. This step distinguishes the present approach from previously reported direct in situ photolithography methods [30, 31, 32, 33] and leads to a substantial improvement in pattern resolution and fidelity by confining MHP precursors to the crosslinked polymer matrix while removing both unreacted monomers and precursors from the uncrosslinked regions.

Moreover, the in situ crystallized MHPs exhibited a pronounced increase in PLQY upon exposure to air, resulting in a more than five‐fold increase relative to the pristine state. This enhancement originates from air‐induced phase redistribution and surface defect passivation, which collectively suppressed nonradiative recombination. As a result of this combined kinetic and chemical control, MHP CCLs exhibited blue uptake of approximately 80% and 98% for the green and red emitters, respectively, along with corresponding CCE values of ∼45% (green) and ∼30% (red) at a thickness of ∼4 µm. These results demonstrate that high blue uptake and efficient color conversion can be simultaneously achieved at sub‐5‐µm thicknesses, substantially thinner than those typically reported for MHP‐based CCLs [15, 34, 35, 36, 37], showing the potential of KC‐ISP for realizing MHP‐based high‐resolution micro‐displays [8, 9].

### 1.1 Kinetically Controlled Direct In Situ Photolithography (KC‐ISP) Process

**FIGURE 1:**
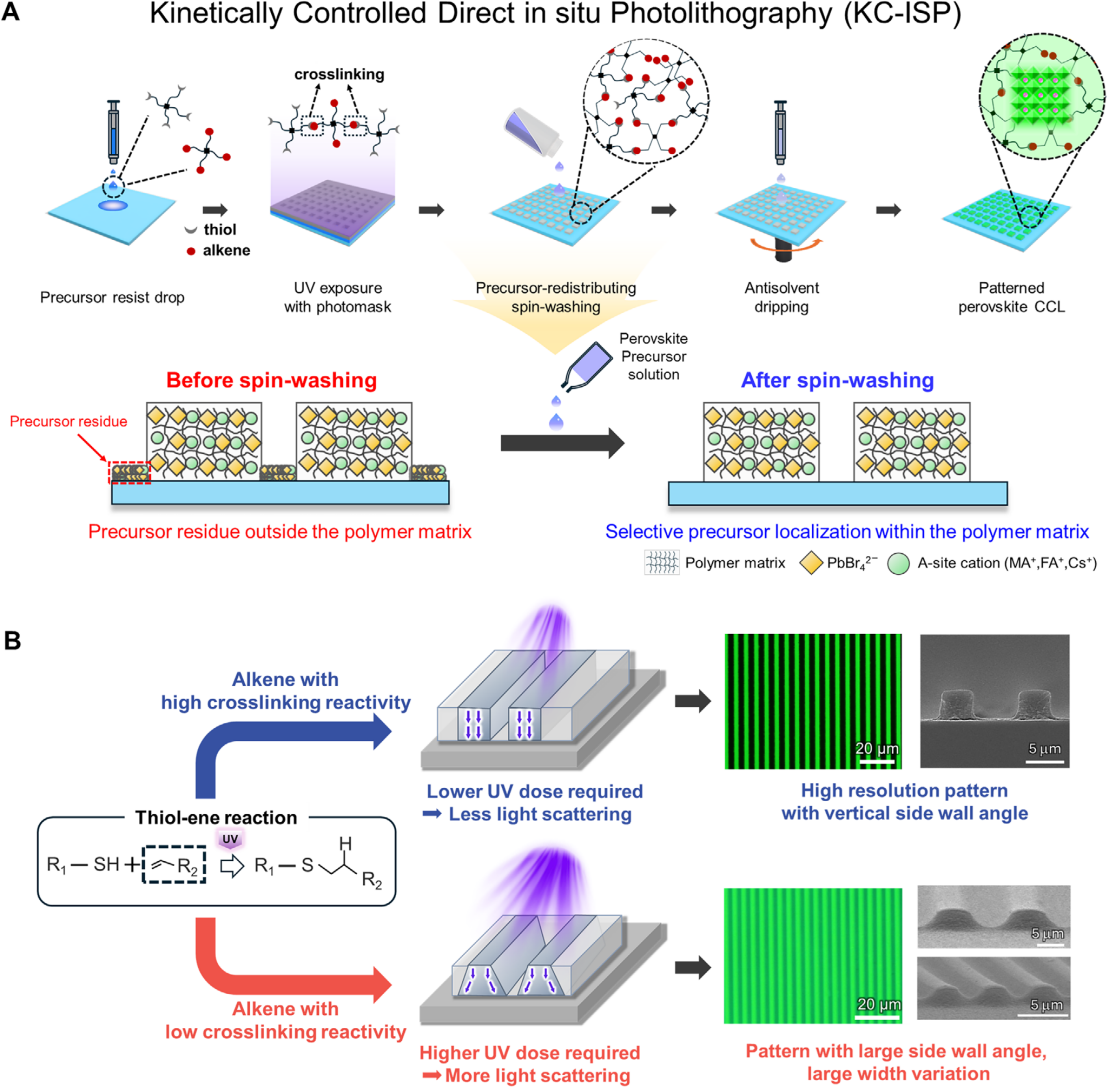
Schematic illustration of (A) KC‐ISP process, (B) the effect of alkene reaction kinetics on pattern resolution and fidelity in KC‐ISP.

Figure 1A illustrates the KC‐ISP process developed in this study. The process begins with casting a precursor resist, consisting of MHP precursors and monomers with thiol and alkene functional groups, onto the substrate. Subsequently, a uniform film is formed by pressing the precursor resist with a photomask, followed by UV exposure, during which thiol–ene crosslinking proceeds selectively in the exposed regions, resulting in the formation of a crosslinked polymer matrix that defines the latent pattern.

After exposure, unreacted monomers (both in the exposed and unexposed regions) are selectively removed through the precursor‐redistributing spin‐washing step using the same solvent employed for the precursor resist, containing only the MHP precursor species. Unlike conventional photolithography, in which unexposed regions remain as a solid photoresist, the unexposed regions in the KC‐ISP process retain a liquid precursor state, which is removed during this spin‐washing step, thereby confining the MHP precursors exclusively within the crosslinked polymer matrix in the exposed regions. The emission color of the resulting patterns can be readily controlled by adjusting the precursor composition at this stage, offering flexible color tunability within a single patterning process.

Following the spin‐washing step, antisolvent treatment, and post‐annealing induce in situ crystallization of MHP domains within the pre‐defined polymer matrix. Unlike previous optical lithography approaches, the MHP emitters in this KC‐ISP process are not directly exposed to harsh lithographic conditions during pattern formation, thereby avoiding potential optical degradation. In this context, since crystallization is confined within the polymer framework, the resolution and fidelity of the resulting MHP patterns closely follow those of the initially defined patterns, underscoring the need for a systematic investigation of thiol–ene reaction kinetics as a key factor in establishing a well‐defined polymer matrix.

### 1.2 Role of Thiol–ene Kinetics in Pattern Resolution and Fidelity

To achieve high‐resolution and high‐fidelity polymer matrix patterning, we focused on the reaction kinetics of the alkene monomers participating in the thiol–ene reaction [38]. In this context, the term “kinetically controlled” is used to describe the dependence of pattern resolution and fidelity on the effective crosslinking reactivity under given exposure conditions. During photopatterning, the degradation of pattern fidelity, such as the formation of slanted sidewalls, is commonly associated with light scattering that occurs at the bottom of the pattern during exposure [39, 40, 41]. Therefore, we hypothesized that incorporating alkene monomers with high reactivity toward crosslinking would enable shorter exposure times and, consequently, mitigate such fidelity degradation.

**FIGURE 2:**
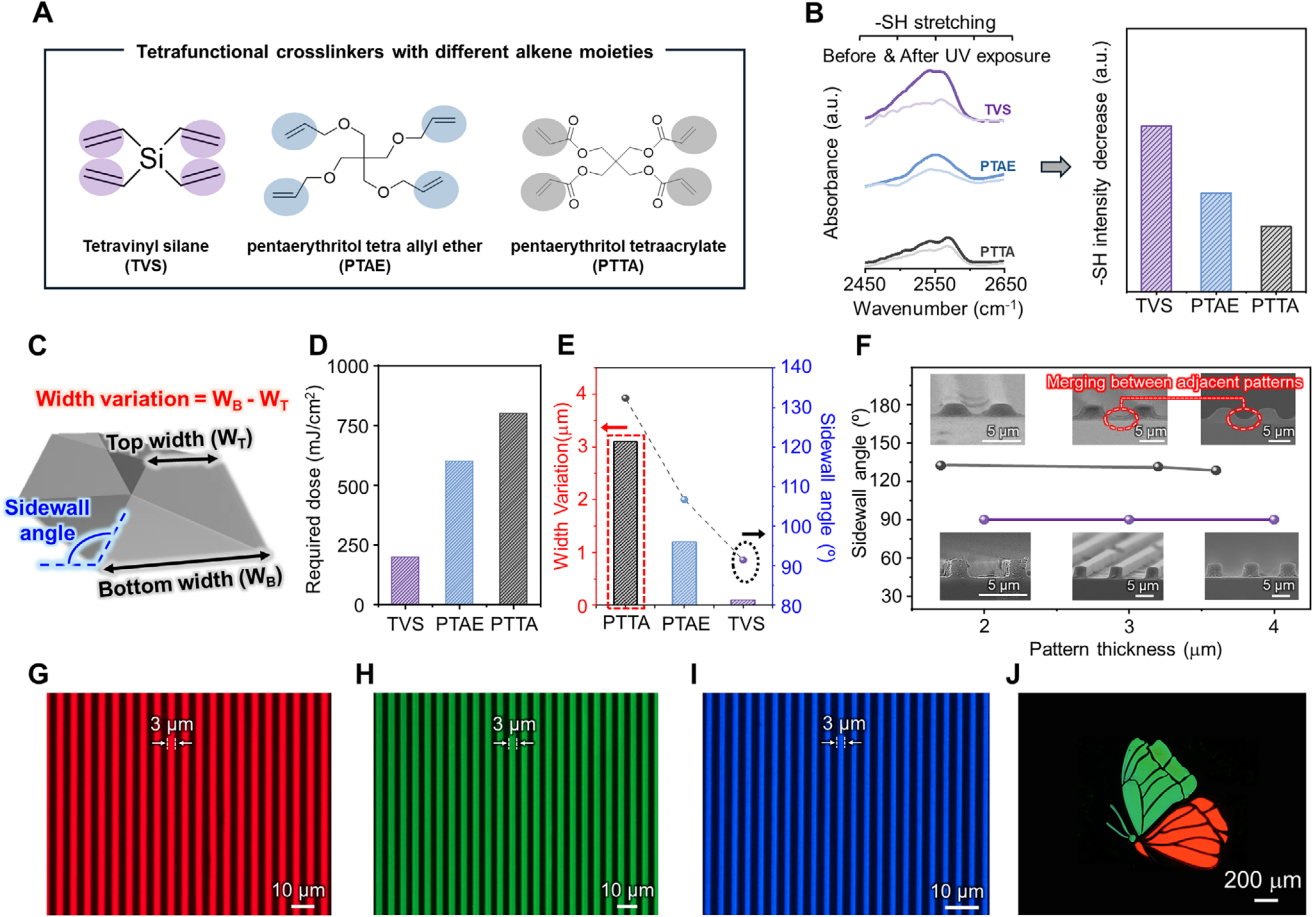
(A) Chemical structures of tetrafunctional crosslinkers with different alkene moieties: TVS, PTAE, and PTTA. (B) FT‐IR spectra showing the decrease in thiol (–SH) stretching intensity before and after UV exposure for different crosslinkers, together with the corresponding quantitative comparison of –SH consumption. (C) Schematic definition of pattern width variation and sidewall angle used for evaluating pattern fidelity. (D) Required UV dose for pattern formation using different alkene crosslinkers. (E) Comparison of width variation and sidewall angle for different crosslinkers (F). Cross‐sectional SEM images showing the dependence of sidewall angle on pattern thickness for different crosslinkers. (G–I) FOM images of red‐, green‐, and blue‐emitting MHP line patterns with a linewidth of 3 µm. (J) Representative FOM image of multicolor MHP patterns fabricated by the sequential KC‐ISP process.

As shown in Figure 1B, consistent with our hypothesis, a clear correlation was observed between the reaction kinetics of the alkene monomers and the resulting pattern fidelity. Monomers exhibiting higher reactivity toward crosslinking require reduced exposure doses, leading to diminished light scattering and the formation of high‐resolution patterns with improved sidewall verticality. In contrast, monomers with lower reactivity require prolonged UV exposure, resulting in larger deviations from vertical sidewalls and, in severe cases, merging between adjacent features. To validate this hypothesis, three tetra‐functional monomers, tetravinyl silane (TVS), pentaerythritol tetraallyl ether (PTAE), and pentaerythritol tetraacrylate (PTTA), were selected as representative systems (Figure 2A).

Raman and electron paramagnetic resonance (EPR) spectroscopy were first employed to confirm that thiol–ene crosslinking is the dominant reaction pathway (Figure S1). Prior to UV exposure, characteristic stretching modes of thiol (2570 cm^−^^1^) and alkene (1590 cm^−^^1^) groups were clearly observed in the Raman spectra (Figure S1A). Upon UV irradiation, the intensities of both stretching modes markedly decreased, indicating consumption of thiol and alkene groups during the crosslinking process [26, 42]. Consistently, EPR measurements reveal the emergence of strong radical signals after exposure, confirming a thiyl radical‐mediated thiol–ene mechanism [26, 43, 44]. To further examine the possibility of competing polymerization pathways, particularly acrylate homopolymerization and disulfide bond formation, control experiments were conducted under identical UV exposure conditions (Figure S2). As shown in Figure S2A, while the mixture of thiol monomer and PTTA underwent successful curing, both thiol‐only and PTTA‐only samples remained in the liquid state without any noticeable curing. In addition, Fourier transform infrared (FT‐IR) spectroscopy shows that the characteristic –SH stretching peak (2527 cm^−^^1^) in the thiol‐only system and the C = C related peak (810 cm^−^^1^) in the PTTA‐only system remain largely unchanged after UV irradiation, indicating that disulfide bond formation and acrylate homopolymerization are negligible under our experimental conditions (Figure S2B).

To quantitatively compare crosslinking kinetics, FT‐IR spectroscopy was performed by monitoring the reduction of the thiol stretching band after exposure to an identical UV dose (Figure 2B). The extent of thiol consumption follows the order TVS (63%) > PTAE (37%) > PTTA (25%), reflecting their intrinsic differences in alkene reactivity. Correspondingly, the minimum exposure dose required for pattern formation increases in the order TVS (∼200 mJ cm^−2^) < PTAE (∼600 mJ cm^−2^) < PTTA (∼800 mJ cm^−2^) (Figure 2D and Figure S2), confirming that curing dose directly reflects crosslinking kinetics.

The impact of these kinetic differences on pattern fidelity was further quantified by analyzing the width variation between the top and bottom regions of the patterns and by measuring sidewall taper angles (Figure 2C). Here, the width variation is defined as W_B_ – W_T_, where W_B_ and W_T_ represent the bottom and top widths of the patterned features, respectively. Positive values therefore, indicate a tapered profile with a wider base than the top. For a consistent comparison, all patterns were prepared with the same thickness (∼2 µm) under otherwise identical processing conditions. As shown in Figure 2E and Figure S3, 2‐µm‐thick patterns fabricated using the TVS‐based resist exhibit nearly vertical sidewalls and negligible width variation. In contrast, decreasing reaction kinetics leads to progressively larger sidewall angles and increased profile distortion. These results indicate that low reactivity toward crosslinking leads to prolonged UV exposure, which enhances unwanted light scattering and promotes lateral curing [39, 40, 41].

To further substantiate this relationship, we investigated the TVS‐based system under varying UV doses (Figure S4). Even for the TVS system with the highest reactivity toward crosslinking, excessive UV exposure deteriorated pattern fidelity. Increasing the exposure dose from 200 to 600 mJ cm^−2^ resulted in a gradual increase in sidewall angle from ∼90° to >130°, demonstrating that kinetic advantages can be negated by overexposure. The degradation of pattern fidelity became more pronounced with increasing pattern thickness (Figure 2F). For TVS‐based patterns, vertical pattern profiles were preserved up to a thickness of 4 µm, whereas PTTA‐based patterns exhibited merging between adjacent patterns beyond ∼3 µm, indicating compromised spatial selectivity.

### 1.3 Precursor Redistribution‐Enabled High‐Resolution and Multicolor Patterning

Precursor redistribution within the polymer matrix enables spatially confined perovskite crystallization, leading to markedly improved pattern resolution and systematic color tunability. Because perovskite crystallization occurs within the polymer framework, inducing crystallization in a kinetically defined and spatially confined matrix is essential for achieving high‐fidelity MHP patterns.

In this context, the precursor‐redistributing spin‐washing step plays a critical role by removing residual precursors and unreacted monomers from the uncrosslinked regions, thereby functioning as a development step while simultaneously confining subsequent perovskite crystallization to the crosslinked polymer matrix. In the absence of this step, conventional direct in situ photolithography yielded substantial background residues and poorly defined features in our system, as evidenced by fluorescence optical microscopy (FOM) images (Figure S6). By contrast, precursor redistribution effectively suppresses MHP formation outside the polymer matrix, resulting in well‐defined patterns with enhanced spatial fidelity (Figure S7).

Beyond improving pattern resolution, this step also enables facile color tuning through controlled variation of the precursor composition. Using this capability, red, green, and blue line patterns with a linewidth of 3 µm were successfully fabricated (Figure 2G–I and Figure S8), corresponding to a resolution exceeding 8,000 pixels per inch (PPI) (assuming a single‐color pixel array). A quantitative comparison across RGB patterns shows that the average sidewall angle and linewidth variation obtained from patterns of each color are comparable. In addition, the defect frequency, evaluated as the number of defects per 100 × 100 µm^2^ area, is similarly low across all RGB patterns, demonstrating consistent and reproducible patterning performance (Figure S9). Various pattern geometries, including hearts, squares, and dots, were also demonstrated (Figure S10), as well as multicolor MHP patterns incorporating thin SiO_2_ interlayers to prevent solvent‐induced damage to underlying layers (Figure 2J and Figure S11). Multicolor patterning without interlayers was also achieved by introducing perovskite nanocrystals as the second layer (Figure S12). In addition, spatial uniformity across the substrate was confirmed, with consistent emission and comparable pattern fidelity observed from the center to the edge regions (Figure S13). By contrast, PTTA‐based patterns exhibit large sidewall angles and pronounced lateral curing, resulting in blurred feature boundaries and reduced pattern contrast in FOM images (Figure S14).

### 1.4 Optical Properties of In Situ Crystallized MHPs

**FIGURE 3:**
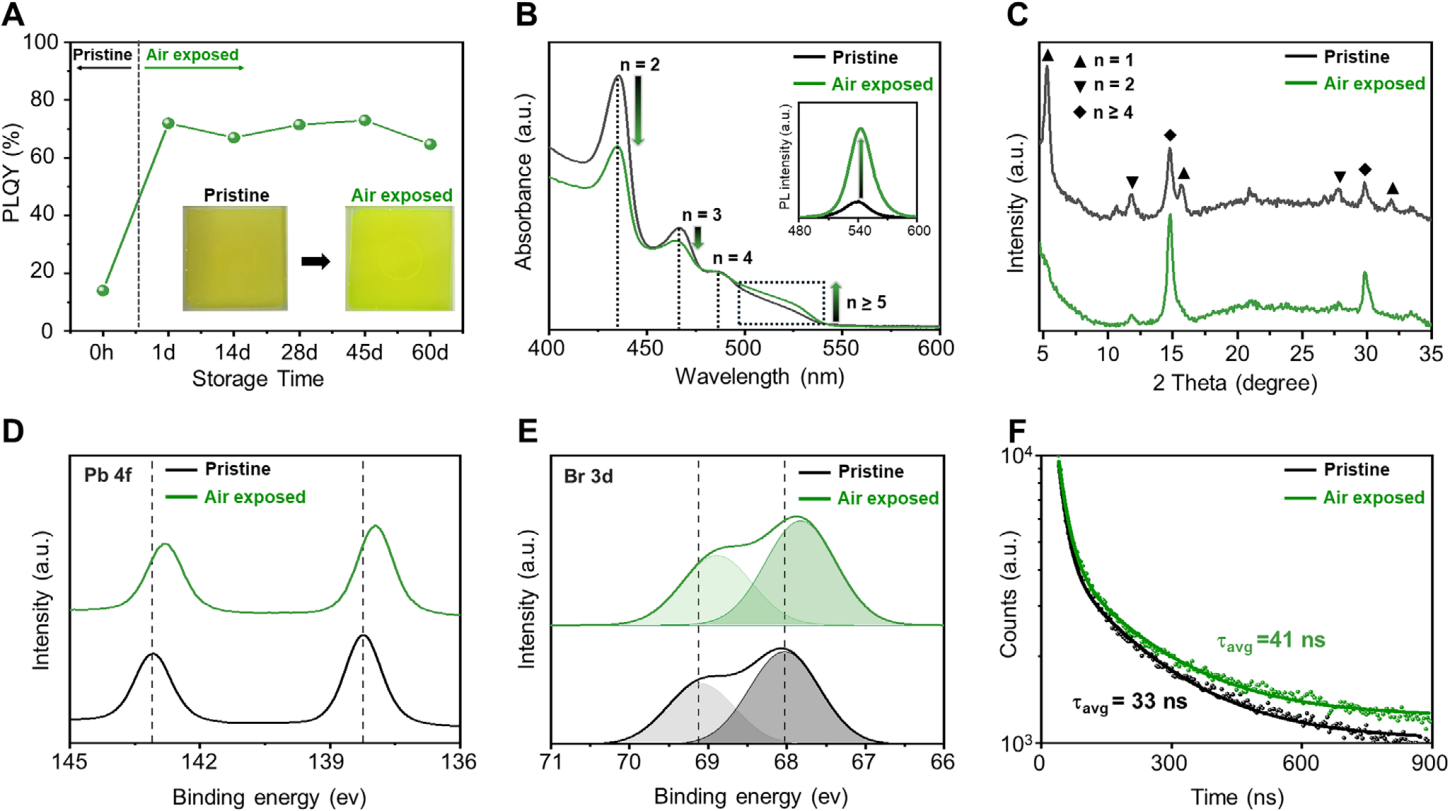
(A) PLQY of pristine and air‐exposed MHP films during storage under ambient conditions with corresponding photographs (inset). (B) UV–vis absorption spectra of pristine and air‐exposed films (inset: PL spectra of each sample). (C) XRD patterns of pristine and air‐exposed films. (D,E) XPS spectra of Pb 4f and Br 3d core levels for pristine and air‐exposed samples. (F) PL lifetime curves of pristine and air‐exposed films.

The optical properties of the in situ crystallized MHPs embedded within the polymer matrix were subsequently investigated (Figure 3). Interestingly, all samples exhibited a significant increase in PL intensity after air exposure (Figure S15), with the PLQY rising more than fivefold, increasing from approximately 14% to about 70% within one day and maintaining its optical properties for over 60 days under ambient conditions (Figure 3A). Across multiple fabricated samples, a consistent trend of significant PLQY enhancement after air exposure was observed (Figure S16). In contrast, the sample stored under N_2_ atmosphere retained its initially low PL intensity and PLQY (Figure S17), indicating that the enhancement originates from air exposure. Given that typical quasi‐2D perovskite thin films generally exhibit PLQY values in the range of ∼60%–70% [45, 46], the relatively low initial PLQY observed in our system suggests that crystallization within the polymer matrix follows a distinct pathway compared to conventional thin‐film processing (Figure S18).

Based on this observation, we systematically conducted a series of analyses to elucidate the underlying mechanism by comparing the as‐prepared samples (“pristine”) with samples exposed to ambient conditions for 24 h prior to measurement (“air exposed”). As shown in Figure 3B, comparison of the UV–vis absorption spectra of pristine and air‐exposed films revealed a decrease in the low‐n quasi‐2D phases (*n* = 2, 3) and a relative increase in higher‐n and 3D‐like phases (*n* ≥ 4) after air exposure [45]. This phase evolution was corroborated by the XRD patterns (Figure 3C), where the pristine samples exhibited distinct diffraction peaks corresponding to low‐n phases, such as *n* = 1 (5.3°, 15.6°, and 31.9°) and *n* = 2 (11.8° and 27.8°). After air exposure, however, the intensities of these low‐n peaks significantly decreased, while those associated with higher‐n phases (*n* ≥ 4, 14.8° and 29.8°) became markedly enhanced [46, 47]. It has been well established that strong exciton–phonon coupling in low‐n phases induces severe nonradiative recombination [48, 49]. Hence, the observed redistribution of n‐phases upon air exposure, shifting from low‐n to higher‐n configurations, can be attributed as a primary factor enabling more efficient radiative recombination and exciton energy transfer. In addition, the slight redshift observed in the PL peak after air exposure supports this interpretation, as the reduced quantum confinement accompanying the transition from low‐n to higher‐n phases leads to a narrower bandgap (Figure S15) [50, 51].

Beyond this phase redistribution effect, previous reports have suggested that O_2_ and H_2_O molecules in air can act as defect passivators for MHPs [52, 53, 54]. To examine this possibility, XPS analysis was performed to evaluate the chemical state variation before and after air exposure. The results revealed that both Pb 4f_7/2_ (138.2 eV) and Pb 4f_5/2_ (143.1 eV) peaks in the pristine sample shifted by approximately 0.3 eV toward lower binding energies after air exposure (Figure 3D). Similarly, the Br 3d_3/2_ (69.1 eV) and Br 3d_5/2_ (68.0 eV) peaks exhibited a negative shift of about 0.2 eV (Figure 3E). These shifts indicate an increase in the local electron density around Pb and Br atoms, implying that O_2_ and H_2_O molecules interact with the MHPs surface and serve as mild defect passivators by donating lone‐pair electrons. Such surface passivation further mitigates nonradiative recombination, synergistically enhancing the optical properties of the in situ crystallized MHPs together with the phase redistribution effect.

To substantiate these findings, time‐resolved photoluminescence (TR‐PL) analysis was performed on both pristine and air‐exposed samples (Figure 3F). The trend in average carrier lifetime exhibited a strong correlation with the PLQY increase: the lifetime increased from approximately 33 ns in the pristine film to 41 ns after air exposure (Table S1). These results collectively provide evidence that (1) air exposure induces phase redistribution toward higher‐n configurations and (2) species present in air, such as O_2_ and H_2_O molecules, act as mild surface passivators that heal halide vacancies, thereby suppressing nonradiative recombination and enhancing the optical properties of the MHPs.

### 1.5 Color‐Conversion Performance of In Situ Crystallized MHP CCLs

**FIGURE 4:**
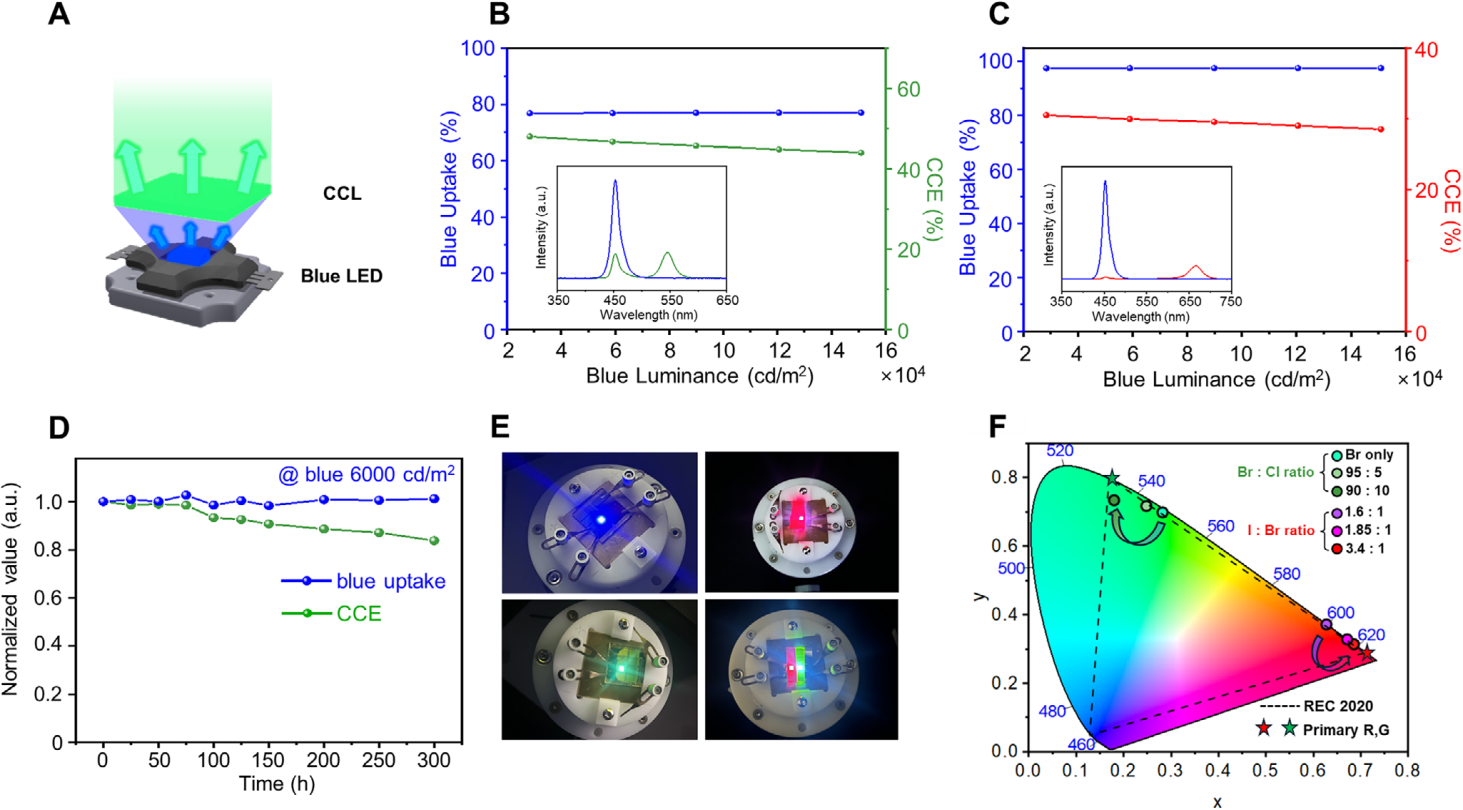
(A) Schematic illustration of the color‐conversion measurement. (B,C) Blue uptake and CCE of the green (B) and red (C) CCLs as a function of incident blue luminance (insets: corresponding emission spectra under blue excitation). (D) Time‐dependent stability of blue uptake and CCE measured under continuous blue excitation (6000 cd m^−^^2^, 0.1 W/cm^2^). (E) Photographs showing the color‐conversion characteristics of the MHP CCLs placed on a blue OLED. (F) CIE 1931 chromaticity diagram of the green and red CCLs with different halide compositions.

We demonstrate that efficient and stable color conversion can be achieved using a thin CCL even under high brightness excitation. To evaluate the color conversion performance of our MHP CCLs, we placed the fabricated CCL (on a glass substrate) directly above a blue LED and measured their blue‐to‐green and blue‐to‐red conversion characteristics (Figure 4A, Figure S19). The film thickness was approximately 4 µm, and the average surface roughness (R_a_) measured by AFM was around 10 nm (Figure S20). The film thickness could be systematically controlled by varying the monomer concentration in the precursor resist, enabling tunability in the micrometer thickness range (Figure S21).

To evaluate the color‐conversion performance of our MHP CCLs, we employed blue uptake and the corresponding CCE as quantitative performance metrics under varying excitation luminance. The blue uptake was defined as the fraction of incident blue photons absorbed by the color‐conversion layer and was calculated by integrating the spectral difference between the reference blue intensity (I_blue_) and the transmitted blue intensity (I*_blue_). The CCE was determined as the ratio of the emitted green (or red) photon flux (I_green_, I_red_) to the absorbed blue photon flux, obtained by integrating the PL spectrum. The explicit equations used for these calculations are provided in Figure S22.

For the green CCL (Figure 4B), as the incident blue luminance increased from ∼2 × 10^4^ to 1.5 × 10^5^ cd m^−2^, the blue uptake remained nearly constant at ∼80% across the entire range. Correspondingly, the CCE also maintained a luminance‐independent value of approximately 45%, showing only a minimal decrease at the highest excitation levels. Here, the excitation intensity is expressed in terms of blue luminance (cd m^−^^2^), which is a standard metric for display operation, while the blue uptake and CCE are evaluated based on photon flux to accurately reflect the photophysical conversion process. It is noted that increasing the MHP content within the polymer matrix further enhances blue uptake, whereas the CCE decreases due to increased self‐absorption, resulting in the highest CCE being observed at a Pb^2+^ concentration of 0.15 m (Figure S23). Meanwhile, as shown in Figure S24, increasing the film thickness from ∼2 to ∼4 µm leads to a gradual increase in blue uptake and CCE, indicating that thicker films enable more efficient absorption and conversion of incident blue light.

Similarly, the red CCL exhibited consistently high blue uptake and stable CCE under identical excitation conditions. As shown in Figure 4C, the red CCL maintained a blue uptake of approximately 98% across the entire luminance range, while the CCE remained around 30% without noticeable variation even at the highest excitation levels. Importantly, these blue‐uptake and CCE values were achieved using a CCL thickness of only ∼4 µm, which is substantially thinner than those typically reported for MHP‐based CCLs (Table S2) [15, 34, 35, 36, 37].

The long‐term operational stability of our MHP CCLs was evaluated by monitoring their blue uptake and CCE under continuous blue excitation (Figure 4D, Figure S25). Over a continuous operation period of ∼300 h, the blue uptake remained essentially unchanged, while the CCE exhibited only a modest decrease, retaining ∼80% of its initial value. Such stable behavior indicates that the emissive MHP domains do not undergo significant photo‐induced nonradiative recombination or structural degradation during prolonged excitation. To visually validate the practical color‐conversion capability and spatial uniformity of the fabricated MHP CCLs, red and green CCLs were placed directly on top of a blue OLED, and the resulting emission photographs were obtained (Figure 4E). The pristine device displayed a uniform blue emission as a reference, whereas integration of the red or green CCL yielded a clear conversion to the corresponding emission color over the device area, confirming effective blue‐light absorption and color conversion without noticeable color mixing.

To quantitatively examine the color tunability of our MHP CCLs, the chromaticity coordinates of the green‐ and red‐emitting films were plotted in the CIE 1931 color space (Figure 4F, Figure S26). For the green CCLs, systematic adjustment of the Br:Cl halide ratio induced a clear shift of the emission coordinates toward the Rec. 2020 primary green point. Increasing the Cl content progressively shifted the green emission to shorter wavelengths, moving the coordinates from the Br‐only composition (*x* ≈ 0.275, y ≈ 0.702) toward more saturated green regions (e.g., *x* ≈ 0.184, y ≈ 0.731 at Br:Cl = 90:10). This compositional control enables precise tuning of the green emission to closely approach the Rec. 2020 gamut boundary. For the red CCLs, the chromaticity was similarly tuned by varying the I:Br ratio. Increasing the iodide proportion led to a gradual redshift of the emission, moving the coordinates from moderately saturated red values (*x* ≈ 0.665, y ≈ 0.334 at I:Br = 1.6:1) toward deeper‐red regions closer to the Rec. 2020 primary red point (*x* ≈ 0.704, y ≈ 0.295 at I:Br = 3.4:1). These results collectively demonstrate the strong compositional tunability of the in situ crystallized MHPs, enabling coverage of a broad portion of the Rec. 2020 color space with both green and red CCLs.

## 2 Discussion

In summary, we established a KC‐ISP process to fabricate high‐resolution and composition‐tunable MHP CCLs for micro‐displays. By systematically engineering the thiol–ene crosslinking kinetics, we achieved rapid polymer network formation that minimizes lateral curing and light‐scattering effects, enabling pattern resolutions down to 3 µm. This represents a significant improvement over prior approaches, where the influence of thiol–ene crosslinking kinetics on pattern resolution was not sufficiently considered. A detailed investigation of the in situ crystallized MHPs revealed that air exposure induces a redistribution from low‐n to higher‐n perovskite phases, accompanied by defect passivation by O_2_ and H_2_O, thereby enhancing the PLQY from ∼14% to ∼70%. When implemented as CCLs, the optimized green and red MHP CCLs exhibited strong blue absorption (∼80% and ∼98%) and practical CCE values (∼45% and ∼30%), with stable optical responses across a broad luminance range and ∼90% retention under extended excitation. This result highlights that efficient and stable color conversion can be realized at sub‐5‐µm thicknesses, a key requirement for high‐resolution micro‐display applications. Additionally, compositional tuning of halide ratios (Br:Cl for green, I:Br for red) enabled precise chromaticity control approaching the Rec. 2020 primaries. Overall, this work provides a comprehensive framework for high‐fidelity patterning and optical optimization of MHP CCLs, offering a promising route toward efficient, wide‐gamut, and high‐resolution micro‐displays.

### 2.1 Materials

Phenethylammonium bromide (PEABr), Formamidinium bromide (FABr, >99.99%), Methylammonium bromide (MABr, >99.99%) were purchased from GreatCell Solar. Cesium iodide (CsI, >99.999%), Lead iodide (PbI_2_, >99%), Rubidium bromide (RbBr, 99.6%), Cesium bromide (CsBr, 99.9%), Pentaerythritol tetrakis(3‐mercaptopropionate) (PTMP, >95%), Tetravinyl silane (TVS, 97%), N,N‐Dimethylformamide (DMF, >99.8%), Dimethyl sulfoxide (DMSO, >99.9%), Chloroform (>99.9%), 5,5‐Dimethyl‐1‐pyrroline N‐oxide (DMPO) were purchased from Sigma–Aldrich. Pentaerythritol Tetraacrylate (stabilized with MEHQ) (PTTA, >80%), Vinyltrimethoxysilane (VTMS, >98%) were purchased from TCI. Pentaerythritol Tetraallyl Ether (PTAE, >94%) was purchased from Fujifilm wako chemical. All of the reagents were directly used without any purification.

### 2.2 Preparation of Precursor Resist and Exchange Precursor Solution

Precursor resist was prepared by dissolving 0.5 mmol MABr, 0.25 mmol PbBr_2,_ 0.5 mmol PTMP, 0.5 mmol alkene monomer (TVS, PTAE, PTTA) into 0.8 mL DMF and 0.2 mL DMSO and was stirred for at least 2 h at room temperature before use. Blue precursor solution was prepared by dissolving 0.33 mmol PEABr, 0.2 mmol RbBr, 0.13 mmol CsBr, PbBr_2_ 0.5 mmol into 1 mL DMSO. Green precursor solution was prepared by dissolving 0.1 mmol FABr, 0.15 mmol PbBr_2,_ 0.1 mmol PEABr into 0.8 mL DMF and 0.2 mL DMSO. Red precursor solution was prepared by dissolving 0.166 mmol CsI, 0.2 mmol PbI2, 0.166 mmol PEABr into 1 mL DMF. All precursor solution was prepared based on a previously reported recipe [45, 55, 56].

### 2.3 Kinetically Controlled Direct In Situ Photolithography Process

All procedures up to the UV exposure step were conducted under atmospheric conditions, while the subsequent steps were carried out under a nitrogen atmosphere. All substrates were treated by VTMS vapor deposition method; a small amount of VTMS was placed in a sealed container with substrates, and heated to 90°C for at least 2 h. After casting 10 µL of precursor resist on the center of the substrate, a pre‐made photomask was contacted with the precursor resist on the substrate to form a film. Subsequently, precursor resist film was exposed to UV light (light source 365 nm, with intensity of 40 mW cm^−2^) to induce thiol‐ene reaction in the exposed areas, leading to the formation of a polymer matrix. After optimal exposure, the substrate was placed on a spin coater and washed with each precursor solution to remove unexposed monomers and tune the precursor composition within the polymer matrix (4000 rpm for 60 sec). During spin‐washing, chloroform was dropped onto the film, followed by annealing with optimal conditions for each precursor composition and MHP patterns were in situ fabricated in the polymer matrix. For multicolor patterns, an intermediate layer (SiO_2_, 100 nm) was deposited between the two layers via electron beam deposition, where the first layer corresponds to the red pattern and the second layer corresponds to the green pattern.

### 2.4 Charaterization Techniques

Scanning electron microscopy (SEM) measurements were performed with SU 5000 (HITACHI). Absorption spectra were measured using a UV–vis spectrophotometer (JASCO V‐770). Fluorescence optical microscopy (FOM) measurements were conducted using a Nikon Eclipse 80i with X‐cite Xylis LED excitation. The photoluminescence quantum yield (PLQY) and photoluminescence (PL) emission spectra were measured using a spectrofluorometer (JASCO FP‐8550) with an integrating sphere. The excitation wavelength for the PL measurement was 465 nm, and the emission wavelength range was 400–800 nm. Timeresolved photoluminescence (TR‐PL) measurements were performed with a Fluorolog‐QM (HORIBA) with 405 nm laser source for excitation. Atomic force microscopy (AFM) measurements were conducted using an NX10 (Park Systems). X‐ray diffraction (XRD) measurements were performed with Ultima IV (RIGAKU). X‐ray photoelectron spectroscopy (XPS) spectra were measured in a nitrogen atmosphere using a K‐alpha (Thermo VG Scientific) with an Al source (1486.7 eV). Fourier transform infrared (FTIR) measurements of the film were conducted on a Nicolet iS50 (Thermo Fisher Scientific), with a range of 4000–400 cm^−^^1^ and resolution of 4 cm^−^^1^, using attenuated total reflection (ATR). Raman spectroscopy measurements were performed with LabRAM HR Evolution Visible_NIR (HORIBA). Electron paramagnetic resonance (EPR) measurement was performed with JES‐FA200 (JEOL). For colorful patterns, SiO_2_ was deposited by electron beam evaporator (Atech). Color conversion efficiency (CCE) was measured by Fiber coupled spectrometer (LE‐5400, Otsuka Electroncis) with an integrating sphere. Blue LED was operated using a source meter (Keithley 2400).

## Author Contributions

M. Hwang designed and conducted the experiments and analyzed the data. J. Jang contributed to a few SEM, XPS measurements and figure composition. J.‐S. Lee contributed to the optimization of the CCL precursor recipes. S. Maeng contributed to EPR analysis and a few FT‐IR measurements. S. J. Park contributed to AFM measurements and figure composition. S. Lee contributed to SiO_2_ deposition (multi‐color patterning) and a few XPS measurements. J. J. Lee contributed to XRD measurements. J. H. Choi and J. K. Kang contributed to EPR measurements. H. Cho fabricated the OLED devices. M. Hwang and H. Cho. wrote the manuscript. H. Cho. supervised the project.

## Conflicts of Interest

The authors declare no conflicts of interest.

## Supporting information




**Supporting File**: smll74360‐sup‐0001‐SuppMat.docx.

## Data Availability

The data that support the findings of this study are available from the corresponding author upon reasonable request.
