# Supplementary material for: Kinetically Controlled Direct In Situ Photolithography of Perovskite Color‐Conversion Layers
Source: Small. 2026 Jul 3;22(47):e74360. doi: 10.1002/smll.74360 (PMC13495818; doi:10.1002/smll.74360)
Supplement: Supplementary file 1 [file SMLL-22-e74360-s001.docx]

Supporting Information for

**Kinetically Controlled Direct in situ Photolithography of Perovskite Color-Conversion Layers**

Mingyu Hwang^1^, Jaedong Jang^2^, Jun-Seo Lee^2^, Seongkyu Maeng^2^, Sun Jae Park^2^, Seungwoo Lee^1^, Jae Jun Lee^2^, Jong Hui Choi^2,3^, Jeung Ku Kang^2^, Hyunsu Cho^4^ and Himchan Cho^1,2,*^

1 Graduate School of Semiconductor Technology, School of Electrical Engineering, Korea Advanced Institute of Science and Technology (KAIST), 291 Daehak-ro, Yuseong-gu, Daejeon 34141, Republic of Korea

2 Department of Materials Science and Engineering, Korea Advanced Institute of Science and Technology (KAIST), 291 Daehak-ro, Yuseong-gu, Daejeon 34141, Republic of Korea

3 School of Materials Science and Engineering, Yeungnam University, 280 Daehak-ro, Gyeongsan 38541, Republic of Korea

4 Reality Display Research Section, Electronics and Telecommunications Research Institute (ETRI), Daejeon, 34129, Republic of Korea

**^*^E-mail**: [himchan@kaist.ac.kr](mailto:himchan@kaist.ac.kr)

**Figure S1.** (A) Raman spectra of the precursor resist measured before and after UV exposure. (B) EPR spectra of the precursor resist recorded with and without UV exposure, and the corresponding simulated spectrum. DMPO was used as a spin trap. We obtained the simulated spectrum of DMPO/S⸱ adduct from an EPR simulator available online, using hyperfine coefficients of aH = 13.78 G and aN =13.26 G.

**Figure S2.** (A) Photographs of precursor resists containing different combinations of monomers (thiol monomer + PTTA mixture, thiol monomer only, and PTTA only) before and after UV irradiation. (B) FTIR spectra of thiol-only (top) and PTTA-only systems (bottom) measured before and after UV exposure.

**Figure S3.** Photographs of precursor resists containing different alkene crosslinkers (TVS, PTAE, and PTTA) before and after UV curing.

 **Figure S4.** Cross-sectional SEM images of patterns fabricated using different alkene crosslinkers: PTTA-, PTAE-, and TVS-based systems (left), and corresponding top (W_T_) and bottom (W_B_) pattern widths extracted from the SEM images (right).

 **Figure S5.** Cross-sectional SEM images of TVS-based patterns fabricated at different UV exposure doses (200, 400, and 600 mJ cm^−^²) (left) and the corresponding sidewall angles plotted as a function of exposure dose (right).

**Figure S6.** Schematic illustration of conventional direct in situ photolithography and corresponding FOM images of patterned MHPs.

**Figure S7.** Photographs illustrating the effect of precursor-redistributing spin-washing step. The entire substrate was first treated with VTMS, after which a polymer matrix was formed on only half of the substrate. The precursor-redistributing spin-washing step was then applied to the whole substrate, resulting in color formation and PL emission exclusively within the polymer-matrix-covered region, while the bare VTMS-treated area remained free of residual MHPs.

**Figure S8.** Schematic illustration of color tunability enabled by the precursor-redistributing spin-washing step in the KC-ISP process.

\

**Figure S9**. Quantitative comparison of patterning performance across RGB compositions. Optical images highlight defect distributions for blue, red, and green patterns, with defect frequency evaluated per 100 × 100 μm² area. Cross-sectional SEM images were used to extract sidewall angles and linewidth variations.

**Figure S10.** FOM images of MHP patterns with different shapes and feature sizes, including square, heart, ring, and array patterns. Scale bars are indicated in each image.

 **Figure S11.** Schematic illustration of a multicolor patterning process.

**Figure S12.** Demonstration of multicolor patterning without an intermediate layer.

**Figure S13.** (A) Evaluation of spatial uniformity across the film. (B) Comparison of pattern formation at different radial positions.

**Figure S14.** FOM image of patterns fabricated using the PTTA-based system.

**Figure S15.** PL spectra of TVS-, PTTA-, and PTAE-based systems measured before and after air exposure.

**Figure S16.** Statistical distribution of PLQY for pristine and air-exposed samples (n = 9).

**Figure S17.** PL spectra (left) and corresponding PLQY values (right) of samples stored under ambient air or nitrogen (N₂) for 1 day.

**Figure S18.** Comparison between conventionally prepared quasi-2D perovskite films and polymer matrix-embedded MHP films.

**Figure S19.** Photographs of the color-conversion measurement setup showing green and red emission from CCLs placed on a blue excitation source.

**Figure S20.** Cross-sectional SEM image showing the thickness of the CCL (left), AFM height image with the corresponding average surface roughness (Ra) value (middle), and three-dimensional AFM topography image (right).

**Figure S21.** Cross-sectional SEM images of films fabricated with different monomer concentrations (0.25 M, 0.4 M, and 0.5 M)**.**

**Figure S22.** Schematic illustration of the definitions and calculation procedures for blue uptake and CCE, based on the integrated spectral intensities of the incident blue emission, transmitted blue light, and converted emission.

**Figure S23.** Blue uptake and CCE as a function of Pb^2+^ concentration in the perovskite CCL.

**Figure S24.** Film thickness-dependent color-conversion performance.

**Figure S25.** Photoluminescence (PL) spectra recorded during the photostability measurement at different illumination times.

**Figure S26.** Normalized PL spectra of green and red CCLs with different halide compositions, corresponding to the chromaticity data shown in Figure 4F.

**Table S1.** Fitting parameter summary of TR-PL decay curves for pristine and air-exposed samples. The TR-PL decay curves were fitted using a biexponential function, where $\tau$ represents the lifetime and $B$denotes the relative contribution of each decay component. The decay curves are decomposed into two components: a fast decay (t_1_) and a slow decay (t_2_). The fast decay component is commonly associated with nonradiative recombination processes related to defect or trap states, whereas the slower decay component is attributed to radiative recombination. The average lifetime (t_avg_) was calculated from the fitted parameters.

**Table S2.** Summary of representative MHP-based CCLs reported in the literature, comparing film thickness, blue-light uptake, and CCE for green (G) and red (R) emitters.

**References for Supporting Information**

[1] Huang, J.; Li, Z.; Zhu, Y.; Yang, L.; Lin, X.; Li, Y.; Wang, Y.; Wang, Y.; Fu, Y.; Xu, W. Monolithic Integration of Full‐Color Microdisplay Screen with Sub‐5 µm Quantum‐Dot Pixels. *Adv. Mater.* **2024**, *36* (45), 2409025.

[2] Gong, T.; Shi, J.; Zhang, Y.; Bai, W.; Xuan, T.; Zhou, T.; Huang, K.; Xie, R.-J. Rare Earth-Doped Perovskite Quantum Dot Microspheres for Micro-LED Displays. *ACS Energy Lett.* **2025**, 10, 5012–5019.

[3] Bae, S. W.; Park, J.; Jang, J.; Hong, S. W.; Kweon, H.; Lee, S. H.; Shin, Y.; Lee, G.; Yang, H.; Kim, J. Rheology-controlled microlithography of exceptionally stable perovskite nanocrystals for microdisplays. *Nano Energy* **2025**, 111326.

[4] Li, Z.; Ge, J.; Wang, C.; Tong, B.; Yang, G.; Zhong, H.; Dong, Y.; Shi, J. Direct In Situ Cascade Photolithography of Perovskite Quantum Dot Patterns with High Light Conversion Efficiency and Enhanced Stability*. ACS Appl. Mater. Interfaces* **2026**, 18, 4100−4110.

[5] Shin, Y.; Suh, H.; Kwon, B.-H.; Park, J.; Cho, H.; Joo, C. W.; Choi, S.; Shin, J.-W.; Kang, C.-m.; Ahn, D. H. Environmentally stable luminescent perovskite nanocrystals passivated and encapsulated by siloxane hybrids enabling reliable color-converted organic light-emitting diodes. *Chem. Eng. J.* **2023**, *474*, 145889.

[6] Shim, H. C.; Kim, J.; Park, S. Y.; Kim, B. S.; Jang, B.; Lee, H.-J.; Kim, A.; Hyun, S.; Kim, J.-H. Full-color micro-LED display with photo-patternable and highly ambient-stable perovskite quantum dot/siloxane composite as color conversion layers. *Sci. Rep.* **2023**, *13* (1), 4836.
